# Enhancing osseointegration and mitigating bacterial biofilms on medical-grade titanium with chitosan-conjugated liquid-infused coatings

**DOI:** 10.1038/s41598-022-09378-4

**Published:** 2022-03-30

**Authors:** Martin Villegas, Yuxi Zhang, Maryam Badv, Claudia Alonso-Cantu, David Wilson, Zeinab Hosseinidoust, Tohid F. Didar

**Affiliations:** 1 School of Biomedical Engineering, Hamilton, ON L8S 4L8 Canada; 2 Department of Mechanical Engineering, Hamilton, ON L8S 4L8 Canada; 3 Department of Chemical Engineering, McMaster University, 1280 Main Street West, Hamilton, ON L8S 4L8 Canada; 4 Department of Surgery, Juravinski Hospital, 711 Concession Street, Hamilton, ON L8V 1C3 Canada

**Keywords:** Biomaterials - cells, Biomedical materials, Implants

## Abstract

Titanium alloys, in particular, medical-grade Ti-6Al-4 V, are heavily used in orthopaedic applications due to their high moduli, strength, and biocompatibility. Implant infection can result in biofilm formation and failure of prosthesis. The formation of a biofilm on implants protects bacteria from antibiotics and the immune response, resulting in the propagation of the infection and ultimately resulting in device failure. Recently, slippery liquid-infused surfaces (LIS) have been investigated for their stable liquid interface, which provides excellent repellent properties to suppress biofilm formation. One of the current limitations of LIS coatings lies in the indistinctive repellency of bone cells in orthopaedic applications, resulting in poor tissue integration and bone ingrowth with the implant. Here, we report a chitosan impregnated LIS coating that facilitates cell adhesion while preventing biofilm formation. The fabricated coating displayed high contact angles (108.2 ± 5.2°) and low sliding angles (3.56 ± 4.3°). Elemental analysis obtained using X-ray photoelectron spectroscopy (XPS) confirmed the availability of fluorine and nitrogen, indicating the presence of fluorosilane and chitosan in the final coating. Furthermore, our results suggest that chitosan-conjugated LIS increased cell adhesion of osteoblast-like SaOS-2 cells and significantly promoted proliferation (a fourfold increase at 7-day incubation) compared to conventional titanium liquid-infused surfaces. Furthermore, the chitosan conjugated LIS significantly reduced biofilm formation of methicillin-resistant Staphylococcus aureus (MRSA) by up to 50% and 75% when compared to untreated titanium and chitosan-coated titanium, respectively. The engineered coating can be easily modified with other biopolymers or capture molecules to be applied to other biomaterials where tissue integration and biofilm prevention are needed.

## Introduction

Indwelling medical implants are highly susceptible to bacterial infections. In particular, *Staphylococcus aureus* and coagulase-negative staphylococci account for 50 to 60% of total prosthetic joint infections^1^. In most cases, the bacteria will form a biofilm on the implant surface^2–4^. A bacterial biofilm is a surface-associated community enclosed in an extracellular matrix (ECM)^2^. The ECM is a self-secreted polymer composed of glycoproteins, extracellular DNA, and polysaccharides; it promotes bacterial adhesion, increases communication between bacteria, and protects the bacterial community^2–5^. Eradicating biofilms from a medical device can be very challenging, partially because bacterial biofilms provide protection from the immune system and antibiotics^2–4^. Moreover, bacteria in the biofilm can be up to 1000 $$\mathrm{X}$$ more resistant to antibiotics than planktonic bacteria^6^, leaving no option but to remove the infected implant through multiple surgeries. Prosthetic joint infection is the leading cause of hip and knee revision surgeries in North America, accounting for 34% of the total knee revision surgeries in Canada^7^ and nearly 40% in the US^8^. In Canada alone, revision surgeries cost up to 2.3 times the cost of the primary surgery, bringing about an estimated annual cost of $42.1 million dollars to the Canadian healthcare system^7^.

In recent years, material science and surface engineering innovations have led to new strategies to prevent biofouling. Among them, liquid-infused surfaces (LIS) inspired by *Nepenthes* pitcher plants have become increasingly popular due to their excellent repellent properties^9–11^. Liquid-infused coatings are composed of a lubricating layer tethered to the substrate of interest via chemical and topographical means^9^. The lubricating liquid is chosen to be immiscible with other liquids, thus creating a repellent layer that prevents interactions with the underlying substrate. Furthermore, these coatings are commonly described as slippery due to their characteristic low water sliding angles (< 5°)^9^. Liquid-infused coatings can be very effective in creating antifouling surfaces for medical devices^10–19^, and can be easily integrated into many material types, including metals such as stainless steel^20^ and titanium^21^.

Preventing implant failure is not limited to preventing contamination and mitigating biofilm formation; the implant must integrate with the surrounding tissue. Proper bone ingrowth is especially important for load-bearing applications, as insufficient interlocking can cause stress-shielding and promote bone loss, which ultimately results in device loosening and failure of the prosthesis. Aseptic loosening is the second leading cause for revision surgeries in Canada, accounting for 17% of total knee and hip revisions^22^. Several biopolymers and biocompatible coatings have been reported on orthopaedic implants to promote osseointegration^23–25^. Chitosan, a biopolymer found in shellfish, has been reported to induce the proliferation of osteoblast cells, mesenchymal cells, and neovascularization in vivo^26^. Furthermore, chitosan-coated substrates are biocompatible, osteoconductive, degradable, and have higher elastic moduli when compared to titanium surfaces, requiring significantly higher forces to detach osteoblast cells from the coated surface^27^.

In the past few years, several biofunctional liquid-infused surfaces have been created. For example, biofunctional LIS on vascular grafts prevented biofouling and thrombin generation while enhancing endothelialization^12–15^. Furthermore, liquid-infused surfaces have been used to enhance biosensor sensitivity^16,19,28^, micropattern cells^29^ and combined with the antibiotics on medical grade stainless steel to create a fail-safe layer to prevent bacterial infections, and thus biofilm formation of the biomaterial^30^. These studies have shown coatings with multiple functionalities through the co-functionalization of LIS with other biological or chemical components^16,19,30^. These coatings retained the repellent properties provided by the LIS layer but demonstrated secondary properties, such as enhanced tissue integration or bactericidal effects, otherwise not witnessed in conventional LIS coatings. However, most of the previous work regarding tissue integration has been focused on (semi)permeable substrates and not monolithic hard substrates, like titanium, that are the material of choice for orthopaedic applications.

In this work, we set out to create a new generation of biofunctional liquid-infused coatings on titanium alloy with high osteoblast affinity to promote cell attachment and proliferation without compromising the antifouling properties of the liquid-infused layer. This was achieved by incorporating a chitosan conjugated liquid-infused coating on a medical-grade titanium alloy. Titanium alloys were chosen due to their widely use in biomedical implants, displaying high moduli, strength^31^, and biocompatibility^32^, as well as their high resistance to degradation, wear, and fatigue^33^. We conceptualize a modular coating with the superior antifouling properties of LIS, which can be modified with different capture molecules for tissue integration which can be adapted and applied on other materials.

## Result and discussions

### Fabrication of Chitosan conjugated lubricant-infused titanium

**Figure 1:**
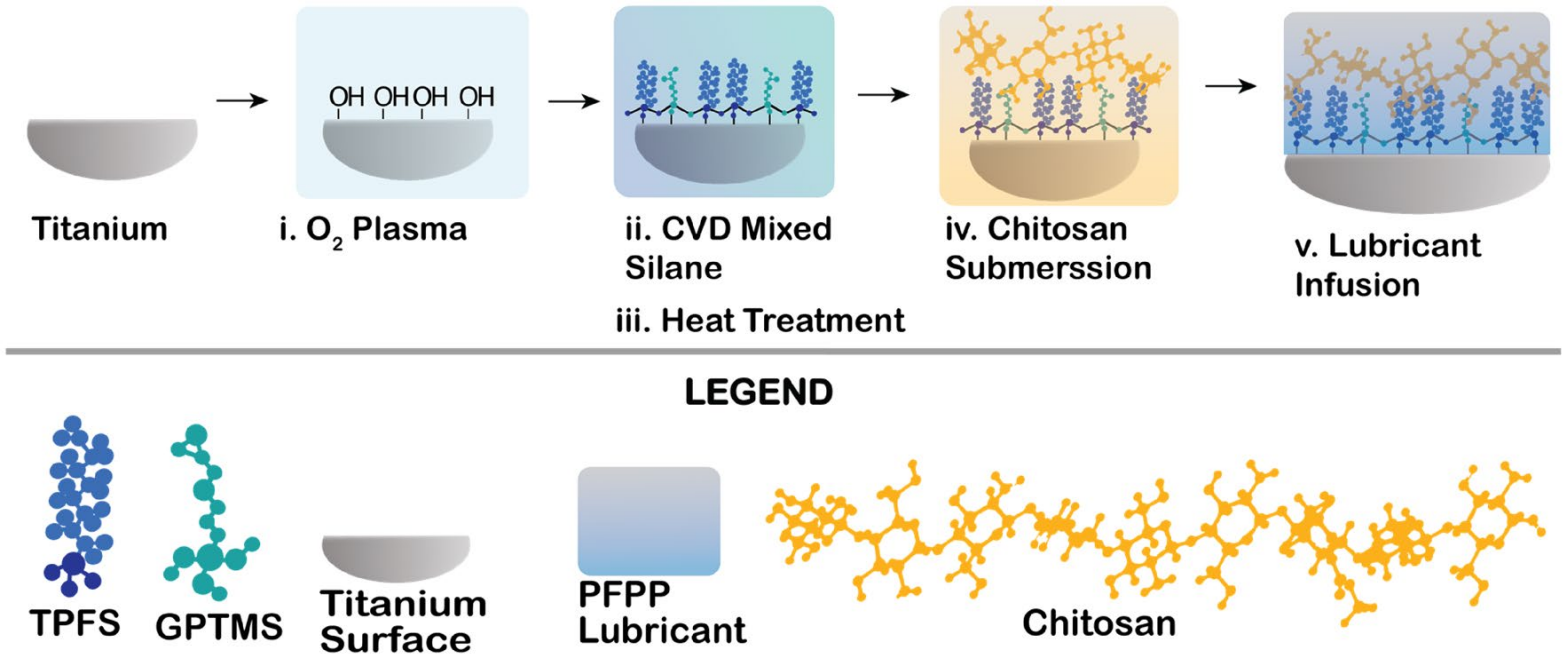
Fabrication schematic. Schematic representation of the fabrication process for the chitosan conjugated liquid-infused coating on titanium.

Medical grade titanium alloy grade 5 (Ti_6_Al_4_V) was functionalized with chitosan and a fluorosilane to create chitosan liquid-infused surfaces (Chitosan–LISs) in the presence of a compatible lubricant. The fabrication of the biofunctional coating is illustrated in Fig. 1. Briefly, cleaned and dried titanium alloys (Ti) were oxygen-plasma treated to hydroxylate and sterilize the surfaces^34^, followed by the chemical vapor deposition (CVD) of a mixture of Trichloro(1H,1H,2H,2H-perfluorooctyl) (TPFS) and 3-Glycidyloxypropyl)trimethoxy (GPTMS) silanes. Mixed silanes were optimized to a ratio of 75:25 for GPTMS and TPFS, respectively. To make the LIS coating, a fluorinated silane (TPFS) was chosen due to its high affinity to the perfluoroperhydro phenanthrene (PFPP) lubricant in order to create a stable interface^41^. It is noteworthy that maintaining a high TPFS availability on the surface is crucial for the liquid-infused coating to remain stable, slippery and repellent^42^. Prior to the chitosan coating, Ti samples were disinfected by submerging the samples in 100% ethanol for a minimum of 20 min and allowed to air-dry inside a biosafety cabinet. Under aseptic conditions, samples were then conjugated with chitosan by submerging the samples in a 50 µg mL^−1^ chitosan solution for 12 h. at 4 ℃. In the past, chitosan has been covalently bonded to titanium using 3-Aminopropyltriethoxysilane/glutaraldehyde^35^ via a two-step reaction or by using triethoxysylilbutyraldehyde (TESBA)^36^. Here, we use an approach similar to the one used by Renoud et al., but replacing TESBA with GPTMS to covalently bond chitosan to the titanium surface. Using GPTMS, we expect the epoxy terminal of GPTMS to react with amine groups in chitosan, while the siloxane terminal of GPTMS would bind to hydroxyl groups on the titanium surface through the reactions shown elsewhere^36–40^. Before testing the samples, substrates were acclimated to room temperature, then, the surfaces were lubricated with 0.5 µL mm^−2^ of PFPP to produce the Chitosan–LIS coating.

### Characterization

**Figure 2:**
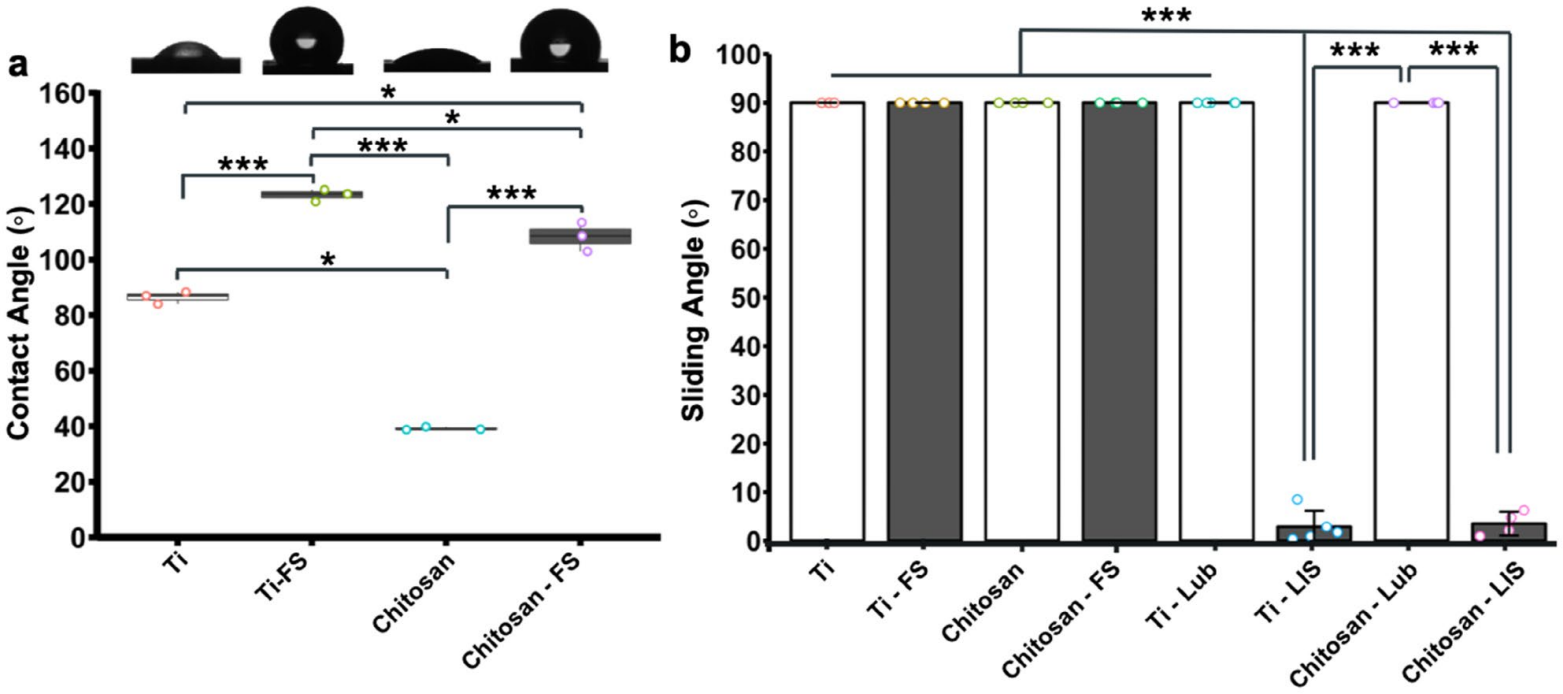
Surface Characterization. (**a**) Water static contact angle measurements on titanium and titanium coated surfaces. The morphology of the water droplet on each surface is shown on top of each bar. Part (a) was analyzed using a Kruskal–Wallis test followed by a Conover’s test to compare the mean rank-sum, with n = 3 for all groups. (**b**) Water sliding angle for the coated titanium surfaces, both bare or lubricated with PFPP. Part (b) was analyzed using a Kruskal–Wallis test followed by a Conover’s test to compare the mean rank-sum, with *n* = 4 for all groups with the exception for Ti-LIS and Chitosan LIS, which had *n* = 5 independent samples. Data points represent the mean, and error bars show the standard deviation. ‘***’ represent a *p*-value of *P* < 0.001, and ‘*’ represent a p-value of *P* < 0.05.

The surface wettability was characterized using contact angle (CA) and sliding angle (SA) measurements. The elemental composition of the surfaces was characterized using X-ray photoelectron spectroscopy (XPS), while the surface roughness and thickness of the coating were measured using an optical profilometer. Contact angles were obtained using an optical goniometer, using 2 µL droplets of Milli-Q water to test the static contact angle. The controlled samples were: untreated titanium alloy (Ti), fluorinated modified titanium (Ti-FS), and chitosan conjugated titanium (Chitosan). The experimental group was the chitosan deposited on fluorinated conjugated titanium (Chitosan-FS). It is worth mentioning that Ti-FS and Chitosan-FS are precursors to their liquid-infused counterparts, Ti-LIS and Chitosan-LIS, whereby they only lack the fluorinated lubricant. As seen in Fig. 2a, fluorinated samples exhibit different wetting properties than their nonfluorinated counterparts. Since hydrophobicity is usually defined at contact angles of 90 degrees, Titanium samples naturally displayed hydrophilic but borderline hydrophobic properties at contact angles of 86.4 ± 2.2°^9^. In contrast, fluorinated titanium samples (Ti-FS) were highly hydrophobic, showing a mean contact angle of 123.2 ± 2.2°(*p*-value = 0.0005 compared to Ti). Similarly, the static water contact angle of chitosan conjugated titanium was highly hydrophilic (39.2 ± 0.6°), while fluorinated conjugated samples containing chitosan displayed hydrophobic contact angles (108.2 ± 5.2°), which dictated a highly significant change (*p*-value < 0.001). Chitosan-FS (108.2 ± 5.2°) samples were more hydrophilic than Ti-FS samples (123.2 ± 2.2°), which was statistically different (*p*-value = 0.034). Overall, the increased contact angles in the samples containing TPFS (Ti-FS and Chitosan-FS) surfaces revealed a successful CVD functionalization process of the fluorinated silane. Furthermore, the fact that Chitosan-FS samples were hydrophobic led us to believe that fluorosilane groups remained present and stable after adding a top layer of chitosan. This is important to maintain a stable LIS coating.

Sliding angles were measured using an off-the-shelf digital angle level. Samples of titanium alloy (Ti), fluorosilane conjugated titanium (Ti-FS), chitosan conjugated titanium (Chitosan), and fluorinated chitosan functionalized titanium (Chitosan-FS) were tested with and without PFPP lubricant. It is worth noting that liquid-infused surfaces require both the PFPP lubricant and the fluorinated chemistry to be stable. Briefly, surfaces were mounted onto the digital angle level, secured using double-sided tape, calibrated to the horizontal plane, and then the surfaces were infused with PFPP lubricant (if needed). The excess lubricant was removed by tilting the surfaces 90 degrees for a minute. Once the surfaces were reset to the horizontal position, a 5 µL droplet of water was pipetted onto the surfaces. The level was raised slowly, and the sliding angle was obtained when the droplets moved. The maximum sliding angle given was 90 degrees; in other words, any droplet pinned on the surfaces past the angle of 90 degrees was given the value of 90 degrees. As witnessed in Fig. 2b, all surfaces lacking a lubricant layer had sliding angles greater than 90°; this included control titanium samples (Ti) as well as titanium coated with chitosan (Chitosan), fluorosilanized titanium (Ti-FS), and titanium conjugated with fluorosilane and chitosan (Chitosan-FS). Lubricated titanium samples (Ti-Lub) did not slide. This can be expected as titanium samples lacking the fluorinated chemistry and were not stable, causing the lubricant to be displaced, causing the water droplet to pin on the surface. In contrast, surfaces that were successfully functionalized with the fluorinated groups and with the addition of the lubricant created a stable liquid-infused layer represented by low sliding angles, which resulted in water repellency and slippery properties. For example, fluorinated-titanium with the addition of the lubricant (Ti-LIS) exhibited slippery properties, depicted by significantly lower sliding angles (3.84 ± 4.33°). Interestingly, samples containing both chitosan and TPFS (fluorosilane) were slippery when lubricated (3.56 ± 4.32°). These results indicate that adding chitosan to previously fluorinated samples does not interfere with the slippery properties to create a liquid-infused surface. Moreover, the results showed no significant difference between Ti-LIS and Chitosan-LIS samples, indicating, again, that the liquid-infused coating was not compromised and behaved similarly when conjugated with chitosan.

Further characterization of the coating was done using X-ray photoelectron spectroscopy (XPS). Figure S1, located in the supporting information, displays the elemental analysis for the coating process of Chitosan-FS at different stages. The percent composition of titanium samples indicates a high level of carbon (33%), oxygen (45%), titanium (10%), and silicon (7%). After the samples were treated with oxygen plasma, the oxygen composition was increased to (49%) while the carbon, silicon, and nitrogen contents were decreased. This change in composition can be expected with surface etching caused by the oxygen plasma and the addition of hydroxyl groups onto the surface. Not surprisingly, none of these samples contained fluorine groups. After the oxygen plasma, the surfaces were coated with a 75:25 ratio of GPTMS and TPFS (Ti-FS-ES). GPTMS is a hydrocarbon silane with an epoxy terminal group, therefore introducing these elements onto the surface. In contrast, TPFS is a fluorocarbon molecule with a silane base. After the conjugation of the mixed silanes, the Ti-FS-ES surfaces display a high fluorine content (37.3%), an increase in silicon (2.5% increase), and a reduction of oxygen (25% decrease) in the elemental composition, thus confirming the dehydration reaction between silanes and hydroxyl groups^12,43^. Interestingly, coating the surface with the mixed silane also removed or blocked any traces of nitrogen on the samples. After the samples were covered with a chitosan layer (Ti-Chitosan samples) on titanium previously treated with the mixed silanes, the samples continued to have high fluorine levels (38%), but nitrogen groups were re-introduced onto the surface. These results indicate the availability of chitosan on the surface since chitosan mainly introduces carbon, oxygen, and nitrogen onto the elemental composition of the surface^44^. Altogether, the XPS and contact angles provide proof of a proper conjugation of the surfaces with fluorine groups and chitosan, while the sliding angles display a stable liquid-infused coating.

The surface topography was characterized using the vertical scanning interferometry mode of an optical profilometer. New samples were coated as previously discussed, and the topography was measured. The results are summarized in Fig. S2 of the supporting information file. Here, untreated titanium (Ti) served as the control group while fully coated samples (lacking the lubricant layer) were used as the experimental groups (Chitosan-FS). The average surface roughness for the samples was 0.69 ± 0.04 and 0.66 ± 0.05 µm for Ti and Chitosan-FS samples, respectively which represents a roughness reduction of only 4% after the addition of the coating. Furthermore, the average surface height of the samples was 5.97 ± 1.10 µm for Ti and 6.47 ± 0.43 µm for Chitosan-FS samples, which represents a height differential of roughly 500 nm, indicating the final thickness of the coating. Even though chitosan-modified samples displayed a decreased average surface roughness and a slightly increased average surface height, these values were not statistically different from untreated Ti samples. Since the coating did not change the topography significantly, no additional mechanical testing was performed.

### SaOS-2 cell adhesion and viability

**Figure 3:**
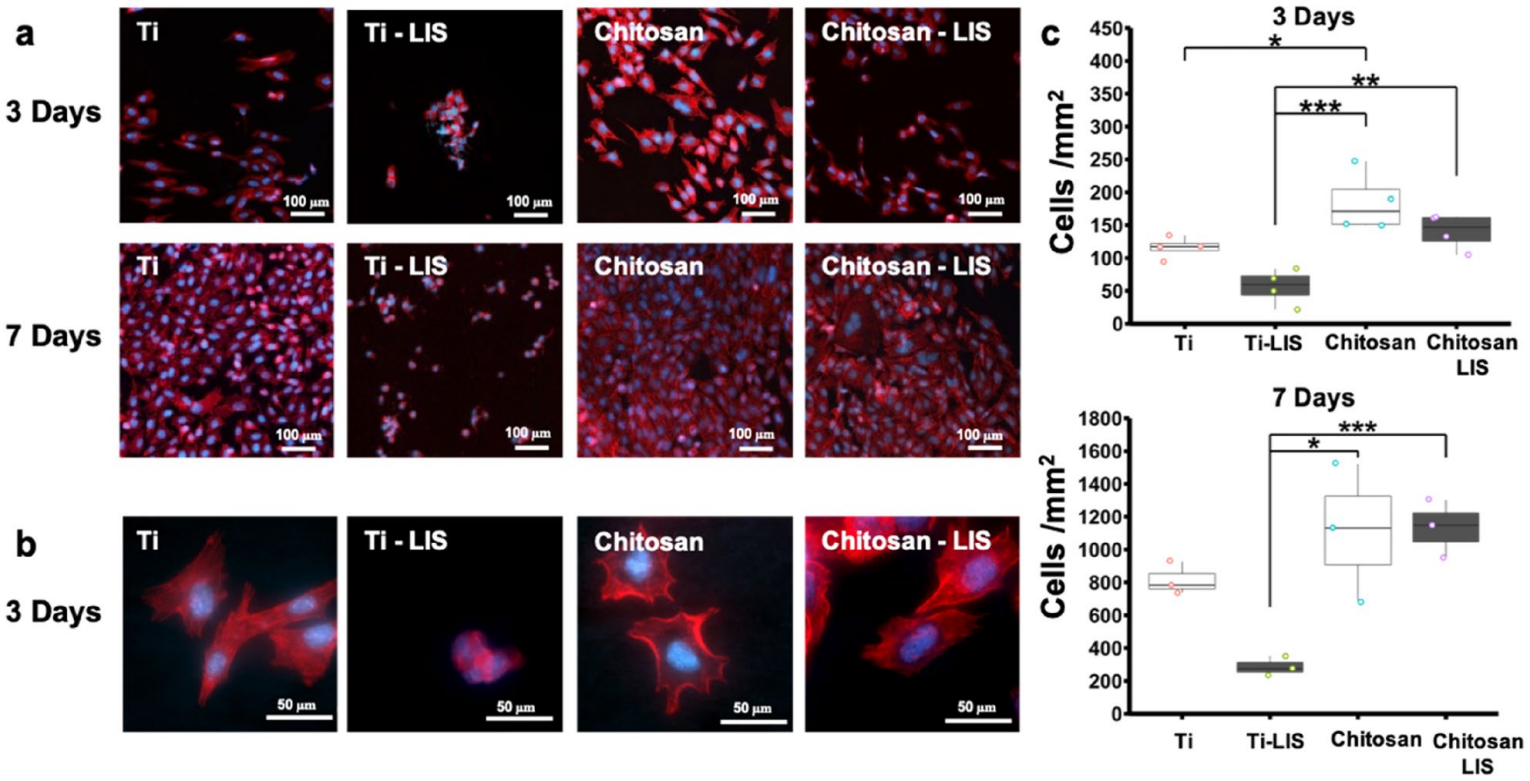
SaOS-2 Cell Proliferation After Three- and Seven-day Cell Cultures. (**a**) Representative fluorescence microscopy *images* (nuclei: blue; microfilaments: red) displaying the density and distribution of adherent cells on titanium and titanium treated surfaces. (**b**) Representative fluorescence microscopy *images* (nuclei: blue; microfilaments: red) displaying the morphology of the cells on titanium and titanium coated surfaces. (**c**) Number of adherent cells on control and treated titanium surfaces after three- and seven-day cell cultures. Error bars represent the standard deviation. *n* = 4 for all groups in the 3-day study and *n* = 3 for all groups in the 7-day study. Statistical analysis was performed using a one-way ANOVA followed by a Tukey test. Significance annotation is shown as ‘*’ for *P* < 0.05, ‘**’ for *P* < 0.01 and ‘***’ for *P* < 0.001.

Mammalian osteoblast-like SaOS-2 osteosarcoma cells were used to model osteoconduction. Cells were cultured for 3 and 7 days. The control groups included untreated titanium (Ti), fluorosilanized coated titanium with lubricant (Ti-LIS), and Chitosan coated titanium (Chitosan) samples. In contrast, samples coated with chitosan on previously fluorosilanized titanium and infused with PFPP lubricant (Chitosan-LIS) were used as the experimental group. Samples were seeded with 100 µL of 8 $\times {10}^{4}$ cells mL^−1^ concentration into each well plus an additional 400 µL of cell media to cover the samples thoroughly. Well-plates were placed in an incubator at 37 ℃ and 5% CO_2_, with culture media being changed every three days. Then, substrates were washed, fixed, stained, and imaged using a fluorescence microscope. Figure 3a shows fluorescent images of the cell densities between control and experimental groups. Here, it is evident that conjugated chitosan samples have high cell densities, which were comparable or superior to titanium samples. In contrast, Ti-LIS samples showed a reduction in cell densities found on the substrate, which was expected due to the repellent nature of the LIS coating. When comparing the cell morphology, Ti-LIS samples showed a clump of cells in a sphere-like configuration (Fig. 3b), denoting poor cell adhesion, which resulted in poor cell proliferation. Moreover, low cell viability is commonly found on LIS samples due to their high repellent properties caused by dissimilar and non-desirable chemistries, which diminish cell adhesion and proliferation, resulting in poor tissue integration in vivo^13,15,45^.

In contrast, Ti samples and all chitosan functionalized surfaces had cells spreading on their surfaces, indicating proper anchoring to the substrate. Proper cell attachment at early stages is indicative of favorable cell behavior, which has been shown to impact cell proliferation and bone formation at later stages^46^. Cell densities were counted using ImageJ, and the results are summarized in Fig. 3c. Here, chitosan-treated surfaces had the highest number of adherent cells. Chitosan samples without LIS coating displayed an average of 185 ± 94 cells mm^−2^ and 1030 ± 419 cells mm^−2^ for the 3- and 7-day incubation periods, respectively. Similarly, Chitosan-LIS surfaces displayed high cell densities of 140 ± 37 cells mm^−2^ and 1133 ± 270 cells mm^−2^ after 3-day and 7-day cultures. The cell densities on Ti control samples were 116 ± 71 cells mm^−2^ for a 3-day incubation period and 828 ± 284 cells mm^−2^ for a 7-days (Fig. 3c). As expected, Ti-LIS substrates without chitosan had the lowest number of adherent cells due to their repellant properties, displaying only 56 ± 71 cells mm^−2^ after 3 days and 185 ± 59 cells mm^−2^ after 7 days of incubation. Cell densities on Chitosan-LIS were not statistically different from Chitosan coated substrates (*p*-value = 0.2 for 3-day and *p*-value = 0.9 for 7 days) or untreated control Ti samples (*p*-value = 0.7 for 3-day and *p*-value = 0.4 for 7-day incubations), indicating that conjugating the LIS coating with chitosan recovers cell adhesion properties comparable to titanium surfaces, which was not observed on Ti-LIS samples. On the other hand, chitosan treated groups displayed significantly higher cell densities compared to liquid-infused coated titanium. When comparing Ti-LIS and Chitosan coatings, the *p*-values were 0.0003 and 0.011 at 3-day and 7-day incubation periods, respectively. Similarly, the *p*-values for Chitosan-LIS coating compared to Ti-LIS were 0.010 and 0.001 for 3-day and 7-day incubation periods, respectively. These results validated our hypothesis, whereby introducing additional biopolymers onto LIS coatings can enhance cell adhesion and thus has the potential to enhance tissue integration with LIS coated surfaces in clinical settings. The mechanism of how the addition of chitosan on liquid-infused coatings increases tissue integration is not fully understood. However, the XPS results in Fig. S1 indicate that chitosan does not completely remove or cover the fluorine groups of the fluorosilane. This was done intentionally as a high concentration of fluorosilane was desired to maintain a stable liquid-infused coating. Nevertheless, the addition of the biological groups found in chitosan, even at low concentrations, can locally change the wettability of the coating and provide anchor points to cells. We hypothesis that the chitosan is potentially creating island-like features where the thickness of the lubricant is significantly reduced due to the dissimilar chemistries offered by the chitosan polymer. Regardless of the mechanism, the results in Fig. 3 prove that the chitosan biopolymer does improve cell adhesion and proliferation when compared to conventional liquid-infused coated titanium (Ti-LIS). The cell culture results presented here are an important stepping-stone and are indicative of a favorable environment for proper bone integration^46^, however, further studies in tissue integration and bone ingrowth, including an animal should be conducted.

### Bacteria repellency properties and biofilm studies

The biofunctional Ti coatings were tested against bacterial biofilm formation to evaluate their bacteria repellent properties. Untreated titanium samples (Ti), conventional liquid-infused titanium (Ti-LIS), and chitosan-coated titanium (Chitosan) were used as control groups. On the other hand, biofunctional samples combining chitosan and liquid-infused coatings (Chitosan-LIS) were used as the experimental coating to evaluate biofilm formation. Briefly, Methicillin-resistant *Staphylococcus aureus* (MRSA) MW2 strain was incubated in Luria–Bertani medium overnight. The next day, the bacteria were diluted at 1:100 bacteria in fresh media and seeded onto newly developed surfaces in a 48-well plate. The bacteria were incubated for 48 h. at 37 ℃ in a shaking incubator at 30 rpm. Afterward, the surfaces were washed with PBS and stained with crystal violet (CV). The CV was dissolved in acetic acid and transferred onto a well-plate to be quantified through light absorbance at 590 nm using a plate reader.

**Figure 4:**
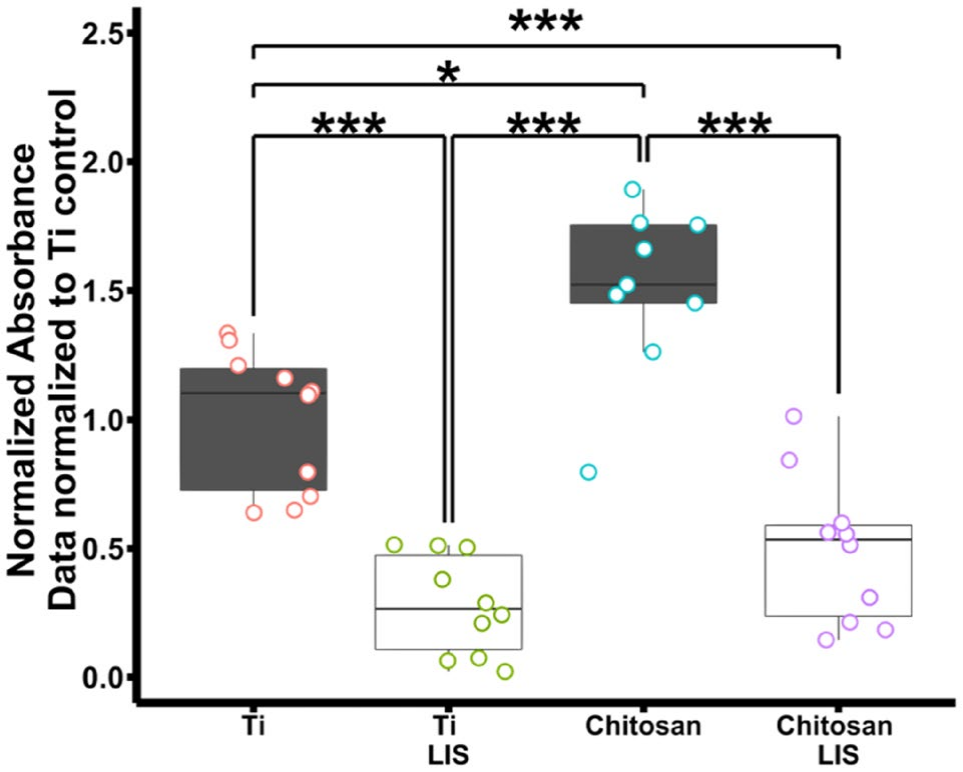
Crystal Violet Evaluation of *S. aureus* Biofilm Formation. Normalized crystal violet absorbance after biofilm growth using MRSA MW2 strain on titanium (Ti) [*n* = 10], liquid-infused titanium (Ti-LIS) [*n* = 10], chitosan-coated titanium (Chitosan) [*n* = 9], and biofunctional chitosan liquid-infused titanium (Chitosan-LIS) [*n* = 10]. The data were normalized by dividing all data points by the mean of the titanium control samples (Ti). Whiskers span the first quartile and fourth quartile range. Statistical significance was tested using Kruskal Wallis, followed by a Conover's test to compare the mean rank-sum. The statistical significance is annotation as ‘*’ for *P* < 0.05, ‘**’ for *P* < 0.01 and ‘***’ for *P* < 0.001.

Biofilm quantities are summarized in Fig. 4. Here, the normalized crystal violet absorbance is shown, where all groups were normalized to Ti (control) samples by dividing each data point by the mean value of the control. Crystal violet values are proportional to the biomass found on the fabricated surfaces; therefore, a high concentration of CV would be associated with a high level of biomass. Similarly, low CV values indicate low biomass. The results indicate that Ti (control) and chitosan-coated samples have high concentrations of biomass. In contrast, both Ti-LIS and Chitosan-LIS samples displayed significantly reduced levels of biomass. The relatively low levels of biomass displayed by Ti-LIS and Chitosan-LIS can be attributed to the excellent bacterial repellency properties, otherwise not seen on chitosan treated samples (*p*-value < 0.001) or Ti (*p*-value < 0.001). In fact, Chitosan-LIS treated samples suppressed bacterial adhesion and biofilm formation to the same extent as liquid-infused modified titanium (Ti-LIS), with crystal violet levels showing no significant difference between these two groups (*p*-value = 0.14). It is noteworthy that chitosan samples had an increased amount of biomass compared to bare titanium surfaces, even though chitosan is believed to have bactericidal properties^47^. This might occur due to an increase in surface anchoring sites, while maintaining chitosan concentration (chitosan concentration in this study 50 µg ml^−1^), below the minimum inhibitory concentration (MIC) for chitosan to have bactericidal properties (previously reported as greater or equal to 1 mg ml^−1^ for *S. aureus*)^48^, therefore, attributing the biofilm reduction of Chitosan-LIS samples solely to the LIS portion of the coating. It is worth noting that liquid-infused coatings do not have bactericidal effects as discussed elsewhere^30^, therefore, if bacteria penetrate the LIS coating and attach to the surface, a biofilm can be produced. In order to prevent such a situation, more research is needed to optimized the coatings to have bactericidal properties and anchoring molecules within the LIS coating to prevent bacteria and provide proper tissue integration at the same time. The mechanism why the Chitosan-LIS coating repels bacterial but allows osteoblast-like cells to attach is not entirely understood, and further investigation is required. However, we hypothesis that the main contributor to this difference can be attributed to the difference in cell size, since osteoblasts are roughly 35 times greater in size than *S. aureus*^49,50^. The increased mass and volume of the osteoblast-like cells can help displace the lubricant from areas where the lubricant-substrate interactions are weakest. In other words, areas where the fluorocarbon is missing due to chitosan deposition, these areas can reduce the forces tethering the lubricant and allow for the attachment of the mammalian cells. In contrast, bacterial cells might not have enough mass to displace the lubricant until enough cells aggregate, therefore resulting in reduced cell attachment and biofilm formation compared to untreated samples lacking the LIS coating (Fig. 4). Overall, these results demonstrate that the addition of chitosan did not negatively impact the bacterial repellent properties offered by the liquid-infused layer, which mirrors similar results previously reported on biofunctional liquid-infused surfaces^15^. These results align with the contact and sliding angle results (Fig. 2) and XPS results (Fig. S1), indicating that Chitosan-LIS possesses stable slippery and repellent properties provided by the fluoridated groups tethering the lubricant layer. Furthermore, the XPS results show the addition of chitosan onto the surface on top of the fluorosilanized coating did not significantly reduce fluorine groups, which can be explained by the addition of the chitosan polymer only to certain spots where the fluorosilane is present and not covering the surface entirely. These findings could be explained by the localized chemical differences, which could aid cells to adhere onto the surface where chitosan was deposited and where the lubricant-substrate interactions are weakest.

Liquid-infused technologies with biomolecules greatly enhance the usability and value of the coating. As discussed earlier, liquid-infused coatings are exceptional at repelling organisms and molecules from the underlying substrate, making them an inappropriate technology where tissue integration is required, as is the case for orthopaedic implants. Combining LIS with osteoconductive biomolecules can overcome this issue by providing anchor points, promoting cell adhesion, and cell proliferating. Although these coatings still require additional investigations to reach clinical settings, here we have shown that adding small concentrations of a biopolymer can greatly increase mammalian cell adhesion and proliferation while not impacting the LIS repellent properties toward bacteria. In other words, combining LIS technology with osteoconductive molecules produces a coating with the potential to prevent the most relevant causes for implant failure, preventing implant infection while creating an environment where the host cells can thrive.

## Conclusion

In summary, biofunctional liquid-infused titanium surfaces functionalized with chitosan improved osteoblast-like cell adhesion and promoted cell proliferation compared to titanium LIS samples. We hypothesize that the chitosan within the LIS layer provided anchor points for cells to spread throughout the titanium device, which demonstrated an increased cell attachment compared to LIS samples, and no significant difference was seen when compared to substrates coated with chitosan alone. Methicillin-resistant *S. aureus* biofilm experiment demonstrated a significant reduction in biomass adhesion on Chitosan-LIS, comparable to traditional liquid-infused coated titanium surfaces, and indicating a suppression of biofilm formation on these surfaces. Furthermore, the contact angle, sliding angle, XPS and biofouling results indicate an active and stable liquid-infused coating on the biofunctional LIS samples. Although the mechanism for mammalian cell attachment on the Chitosan-LIS coating is not fully understood, it is expected that the chitosan created island-like protrusions for cells to attach to when the lubricant is displaced, however, small bacterial cells may not have had enough mass to displace the lubricant from these protrusions. We hypothesize that this fabrication process can be easily adapted for other materials, like cobalt-chromium alloy, carbon fiber, aluminum oxide, and ceramics used in the orthopaedic or related fields. This coating can be used to prevent the formation of biofilms and promote osteoconductive properties for rapid osseointegration between the implant and the tissue.

## Methods

### Materials

Titanium alloy (Grade 5, Ti_6_Al_4_V) sheets (1.6 mm × 30.5 cm × 30.5 cm) were purchased from McMaster-Carr USA (www.mcmaster.com) and cut by CIM Metals (www.cimmetals.com) into 7 mm or 10 mm diameter discs using a water jet. It is worth noting that 7 mm substrates were used for cell density and XPS experiments, while 12 mm samples were used for contact and sliding angle as well as biofilm formation experiments. Titanium substrates were cleaned as described below, but no further mechanical manipulation, grinding, or polishing was performed before or during the coating process for all experiments. Trichloro(1H,1H,2H,2H-perfluorooctyl) silane (TPFS) 97%, perfluoroperhydrophenanthrene (PFPP), (3-Glycidyloxypropyl)trimethoxysilane (GPTMS) 98%, Triton™ X-100 Surfact-Amps™ Detergent Solution, phosphate-buffered saline (PBS), bovine serum albumin (BSA), penicillin/streptomycin, medium molecular weight (190,000–310,000 Da) chitosan and fetal bovine serum (FBS) were purchased from Sigma − Aldrich (Oakville, Canada). McCoy’s 5A modified medium, Trypsin–EDTA (0.25%), methanol-free formaldehyde, and DAPI (4′,6-diamidino-2-phenylindole) were purchased from Thermo Fisher Scientific (Waltham, MA, USA). Phalloidin FITC Reagent was purchased from Abcam (abcam.com).

### Preparation of liquid-infused coating on titanium substrates

Titanium samples were cleaned with acetone, 100% ethanol, and deionized water in sequence in an ultrasonic bath for 10 min. These sonication steps were repeated 3 times with fresh acetone, ethanol, and deionized water. Afterward, samples were dried on a hot plate at 80 ℃, and then titanium samples were placed in a petri dish and placed in an oxygen plasma cleaner (Harrick Plasma Cleaner, PDC-002,230 V) at high power (29.6 W) using medical-grade oxygen at 480 mTorr. Low-pressure oxygen plasma was applied for 10 min to hydroxylate and sterilize the surfaces^34^. Afterward, plasma-treated substrates were placed in a vacuum chamber alongside a petri dish with TPFS (300 µL). The chemical vapor deposition (CVD) of TPFS was carried out for 3 h, at room temperature under vacuum at − 0.01 MPa, followed by an overnight heat treatment at 60 ℃ on a hot plate to finish the reaction. After the heat treatment, salinized surfaces were placed inside a vacuum chamber and placed in a vacuum (with the outlet valve open) for 10 min to remove unbonded silane molecules. Before performing the experiments, samples were submerged in 100% ethanol for 20 min, allowed to air-dry inside a biosafety cabinet to maintain sterile conditions, and infused with PFPP lubricant (0.5 µL mm^−2^$$)$$ to create the LIS.

### Preparation of biofunctionalized liquid-infused chitosan conjugated coating using mixed silanes

Titanium surfaces were cleaned using the steps in the previous procedures. Given that GPTMS can provide anchors for bioconjugation with chitosan and TPFS can maintain repellency of surface, GPTMS and TPFS were used to treat titanium surface during the CVD process. After cleaning the titanium substrates, samples were hydroxylated and sterilized using oxygen plasma (Harrick Plasma Cleaner, PDC-002, 230 V) for 10 min (480 mTorr at 29.6 W). After surface activation, samples were placed in a desiccator and CVD treated with a 75–25% volume ratio of GPTM and TPFS silanes, respectively, for a total volume of 200 µL. The silanes were placed on two separate glass slides located adjacent to the samples in the desiccator. The CVD treatment was carried out for 5 h. at room temperature and a vacuum of at − 0.01 MPa. Then, the samples were placed on a hot plate at 60 ℃ overnight to finish the reaction. Unbonded silane molecules were removed by 10 min post-vacuum in a desiccator and placed in 100% ethanol for a minimum of 20 min. Prior to the chitosan conjugation, samples were taken out of the ethanol inside of a biosafety cabinet and allowed to air dry. Samples were handled in aseptic conditions for future steps.

A 50 µg ml^−1^ of chitosan solution was created by dissolving medium molecular weight (190,000–310,000 Da) chitosan (Sigma Aldrich, Oakville, Canada) in deionized water with 1% acetic acid by volume solution. The solution was continuously stirred at 600 rpm and heated at 40 ℃ for 30 min. Titanium surfaces functionalized with the mixed silanes monolayer were put in a 48-well plate, and chitosan solution (500 µL) was added to each well, covering the samples completely. The well plate was kept at 4 ℃ for 12 h. to complete the bioconjugation, after which, samples were washed with PBS twice and stored at 4 ℃ until needed. Before the cell cultivation experiment, samples were moved from the fridge to the incubator, which allowed samples to acclimate to 37 ℃. Then, PFPP lubricant (0.5 µL mm^−2^) was added to each sample before performing the experiments.

### Contact and sliding angle measurements

Contact angle and sliding angle measurements were used to test the surfaces' wettability. Contact angle measurements were performed using a Future Digital Scientific OCA20 goniometer (Garden City, NY). Samples were tested with a 2 µL droplet of Milli-Q water at room temperature. A minimum of three samples were tested for each group.

The sliding angles were measured using a digital angle level (ROK, Exeter, UK). The apparatus was first calibrated to a horizontal leveled surface, and then samples were placed on the apparatus, followed by the addition of PFPP lubricant. The excess lubricant was removed by tilting the samples vertically for a minute. Once repositioned, a 5 µL droplet of deionized water was placed on the lubricated samples. The tilting stage was raised gently, and the sliding angle was taken as the smallest angle of inclination when the droplet started to slide. If the droplet failed to slide past the angle of 90°, the sliding angle was recorded as a maximum of 90°. The measurements were repeated 3 times on each sample, and a minimum of three samples were used in each group tested.

### X-ray photoelectron spectroscopy

The elemental fraction percentages were obtained from the X-ray photoelectron spectra of newly prepared samples of titanium (Ti), oxygen plasma titanium (Ti-O_2_), titanium coated with the TPFS/ GPTMS mixed silanes (Ti-FS-ES), and chitosan modified samples previously coated with the mixed silanes (Ti-Chitosan). The XPS spectra were obtained using a Physical Electronics (PHI) Quantera-II XPS Microprobe spectrometer. The survey spectra were produced with a monochromatic X-ray source at 25 W using a voltage of 15 kV and a pass energy of 224 eV. All spectra were obtained with a take-off angle of 45° and a step size of 0.8 eV for the survey and 0.1 eV for elemental data.

### Surface Profilometry

Surface average roughness (Ra) and average height (Rz) were obtained using the vertical scanning interferometry (VSI) mode of a Wyko NT1100 optical profilometer (Veeco Corp.) with Vision32 software (Version 2.303).

### Cell density testing of modified titanium surfaces

SaOS-2 osteosarcoma cells (ATCC®) were cultured in mixed McCoy’s modified 5A media supplemented with 15% fetal bovine serum (FBS) and 1% penicillin/streptomycin. SaOS-2 cells were cultivated in an incubator at 37 ℃ and 5% CO_2_, with their media being changed every 3 days. When cells became confluent, cells were detached using Trypsin–EDTA and diluted to a specific concentration for *in-vitro* studies. Modified surfaces without lubricant, LIS modified titanium, and untreated titanium samples were used as control groups, while Chitosan-LIS conjugated samples were taken as the experimental group. The samples were placed into 48 well-plates, followed by adding 400 µL cell media to cover samples completely. Then, 100 µL of 8 $\times {10}^{4}$ cells mL^−1^ SaOS-2 in media was added to each well (concentration was counted by hemocytometer). The well plates were put in an incubator at 37 ℃ with cell media changed every 3 days. After culturing for a specific culture time (3 or 7 days), samples were washed with PBS twice to remove unattached cells. Adherent cells were fixed, permeabilized, and blocked using 4% formalin, 0.1% Triton X-100 and 4% BSA solution, respectively. Finally, DAPI and phalloidin solutions were used to stain the cell nuclei and actin filaments. The surfaces were imaged with a fluorescence microscope to evaluate the cell's density and morphology. The concentration of cell adhesion and proliferation of each sample was calculated using ImageJ software (version 1.53 k Java version 1.8.0_172). Each group had at least 3 samples, and 3 images were taken per sample at random locations and used for ImageJ analysis. The quantification of cell density on each sample was calculated by dividing the total number of cells by the sample's surface area in the image.

### Biofilm formation

Biofilms were grown on surfaces to test the biofilm formation, and adhered biomass was visualized by crystal violet (CV) staining using *Staphylococcus aureus* strain MW2. In each type of surface, 2 samples were used as blank. *S. aureus* was first cultured in Luria–Bertani medium (LB) overnight. Then, Bacteria were diluted at 1:100 bacteria in fresh media and cultured for 2.5 h. until it reached ~ OD_600_ = 0.4, measured by plate reader. Then bacteria were seeded with TSBC5 media (Tryptic Soy broth with 0.5% glucose, 0.2% sodium citrate, 0.6% yeast extract) at a 1: 50 ratio and added to a 48 well-plate covering the samples completely. 35 µL of the mixed solution was put in each well and incubated at 37 ℃ in a shaking incubator at 30 rpm. After a 48-h bacteria culture with *S. aureus*, samples were gently washed in PBS solution and put into a clean 24 well-plate, so that biofilm attached to the wells did not affect the crystal violet results. Then, crystal violet (700 µL) was added for 15 min to stain the samples. Excess CV was removed by a series of wash using DI water until the removed water appeared clear from CV. Samples were allowed to air-dry overnight at room temperature. The next morning, acetic acid (700 µL of 30%) was added to each well for 20 min, allowing the CV to dissolve into the acid solution completely. Solutions were mixed with the pipette. Once the solution was homogeneously mixed, 250 µL solution was transferred to a 96 well-plate. The absorbance of CV was measured at 590 nm using a microplate spectrophotometer (Bio-Rad Laboratories, CA, USA). Finally, optical density values of test and control groups were corrected by background subtraction of absorbance values from blank samples (cultured without bacteria).

### Statistical analysis

All statistical analyses were performed using the open-source software R, version 3.3.2 (www.r-project.org). Nonparametric data were tested with Kruskal–Wallis Rank Sum Test from the R package stats version 3.3.2, followed by a multiple comparison test from R package pgirmess version 1.6.5. Parametric data were tested using either an ANOVA followed by a posthoc Tukey test or an unpaired student t-test. Significance levels were defined as significant ‘*’ at *p*-values of < 0.05, highly significant ‘**’ at *p*-values < 0.01 and as extremely significant ‘***’ with *p*-values < 0.001.

## Supplementary Information

Supplementary Information.
