# Supplementary material for: Enhancing osseointegration and mitigating bacterial biofilms on medical-grade titanium with chitosan-conjugated liquid-infused coatings
Source: Sci Rep. 2022 Mar 30;12:5380. doi: 10.1038/s41598-022-09378-4 (PMC8967836; doi:10.1038/s41598-022-09378-4)
Supplement: Supplementary file 1 — Supplementary Information. [file 41598_2022_9378_MOESM1_ESM.docx]

**Supporting Information**

Enhancing Osseointegration and Mitigating Bacterial Biofilms on Medical-Grade Titanium with Chitosan-Conjugated Liquid-Infused Coatings

Martin Villegas^1,ψ^, Yuxi Zhang^2,ψ^, Maryam Badv^1^, Claudia Alonso-Cantu^3^, David Wilson^4^, Zeinab Hosseinidoust^1,3^*, Tohid F. Didar^1,2^*

^1^School of Biomedical Engineering, ^2^Department of Mechanical Engineering

^3^Department of Chemical Engineering, McMaster University 1280 Main Street West,

Hamilton, ON, Canada L8S 4L8

^4^Department of Surgery, Juravinski Hospital 711 Concession Street Hamilton, ON, L8V 1C3

^ψ^ These authors contributed equally to this work.

*E-mail: [didar@mcmaster.ca](mailto:didar@mcmaster.ca), [doust@mcmaster.ca](mailto:doust@mcmaster.ca)

**Figure S1- X-Ray Photoelectron Spectroscopy.** Percent composition of samples of titanium (Ti), titanium after oxygen plasma (Ti-O2), titanium after CVD deposition of Trichloro(1H,1H,2H,2H-perfluorooctyl) silane and 3-Glycidyloxypropyl)trimethoxysilane (Ti-FS-ES), and titanium coated with chitosan after CVD deposition of mixed silane (Ti-Chitosan). n=3 for all groups with the exception for Ti-Chitosan (n=4), and each sample was measured at three different locations.

**Coating Thickness.**

To investigate the thickness of the coating, new samples were created and tested with the vertical scanning interferometry mode of an optical profilometer. Samples containing chitosan and a layer of fluorosilane do not present a statistical difference of surface average roughness (Ra) or surface average height (Rz) compared to titanium control samples as shown in Figure S2.


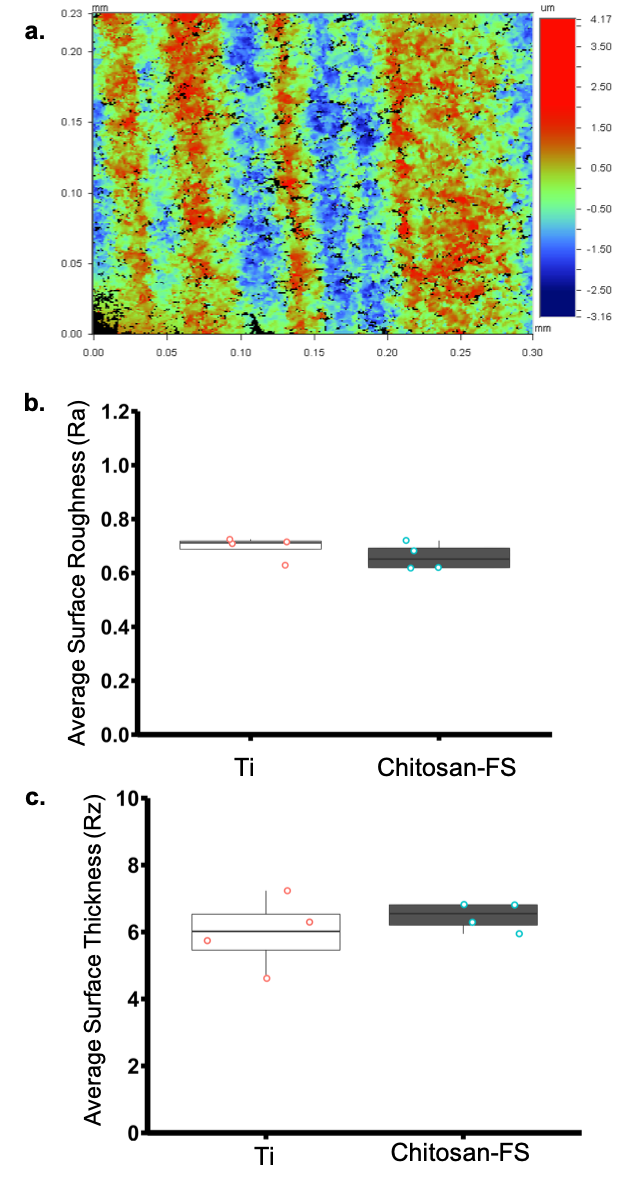


**Figure S2- Average Surface Thickness and Roughness. a.** Representative profilometric image of titanium sample. **b.** Average surface roughness (Ra) for titanium and titanium coated with chitosan and fluorosilane. **c.** Average surface thickness (Rz) for titanium and titanium coated with chitosan and fluorosilane. Samples were analyzed with a paired t-test. n = 4 for all samples. No statistical difference was found between groups.
